# Periodontitis, Metabolic and Gastrointestinal Tract Diseases: Current Perspectives on Possible Pathogenic Connections

**DOI:** 10.3390/jpm12030341

**Published:** 2022-02-24

**Authors:** Dorin Nicolae Gheorghe, Adrian Camen, Dora Maria Popescu, Cerasella Sincar, Allma Pitru, Claudiu Marinel Ionele, Flavia Mirela Nicolae, Claudia Monica Danilescu, Alexandra Roman, Cristina Florescu

**Affiliations:** 1 Department of Periodontology, Faculty of Dental Medicine, University of Medicine and Pharmacy of Craiova, 200349 Craiova, Romania; dorinngheorghe@gmail.com (D.N.G.); flavia.nicolae23@yahoo.com (F.M.N.); 2 Department of Oral Surgery, Faculty of Dental Medicine, University of Medicine and Pharmacy of Craiova, 200349 Craiova, Romania; 3 Department of Periodontology, Faculty of Medicine, “Dunarea de Jos” University, 800201 Galati, Romania; cerasella.sincar@ugal.ro; 4 Department of Oral Pathology, Faculty of Dental Medicine, University of Medicine and Pharmacy of Craiova, 200349 Craiova, Romania; allmapitru75@yahoo.com; 5 Department of Gastroenterology, Faculty of Medicine, University of Medicine and Pharmacy of Craiova, 200349 Craiova, Romania; ioneleclaudiu@gmail.com (C.M.I.); monica.danilescu@gmail.com (C.M.D.); 6 Department of Periodontology, Faculty of Dental Medicine, “Iuliu Hatieganu” University of Medicine and Pharmacy Cluj-Napoca, 400012 Cluj-Napoca, Romania; veve_alexandra@yahoo.com; 7 Department of Internal Medicine and Cardiology, Faculty of Medicine, University of Medicine and Pharmacy of Craiova, 200349 Craiova, Romania; tohaneanu67@yahoo.com

**Keywords:** periodontitis, systemic conditions, diabetes mellitus, hepatic diseases, oral cancer, gastric cancer

## Abstract

Comprehensive research conducted over the past decades has shown that there is a definite connection between periodontal and systemic conditions, leading to the development and consolidation of the “periodontal medicine” concept. The 2018 classification of periodontal conditions uses this concept as a key element of the precise diagnosis of and individualized therapeutical protocols for periodontitis patients. The topic of this review is the pathogenic connections that exist between periodontal disease and metabolic/digestive tract conditions. It is important to remember that the oral cavity is a key element of the digestive tract and that any conditions affecting its integrity and function (such as periodontitis or oral cancer) can have a significant impact on the metabolic and gastrointestinal status of a patient. Thus, significant diseases with links to metabolic or digestive disruptions were chosen for inclusion in the review, such as diabetes mellitus, hepatic conditions and gastric cancers. Periodontal pathogenic mechanisms share several significant elements with these conditions, including mutual pro-inflammatory mediators, bacterial elements and genetic predisposition. Consequently, periodontal screening should be recommended for affected patients, and conversely, periodontitis patients should be considered for careful monitoring of their metabolic and digestive status.

## 1. Introduction

Periodontal disease is one of the most commonly encountered conditions of the oral cavity and dental apparatus and is gaining increasing awareness from both patients and general practitioners. Thus, periodontology has set itself as one of the main specialties of dental medicine [1]. The field of periodontology is focused on the research and comprehensive understanding of periodontal diseases’ pathogenic mechanism, complementary risk factors and clinical features, so as to provide reliable tools for an accurate diagnostic and a predictable treatment plan [1]. 

Periodontal disease is an inflammatory disease caused by the subgingival accumulation of bacterial biofilm [2]. This leads to the inflammation of the gingival tissues (gingivitis) and of the deeper structures of the periodontium (periodontitis). A vast range of local and systemic risk factors are involved in the pathogenic mechanisms of periodontal disease, causing an individualized immune response to the bacterial biofilm and a diverse clinical manifestation from patient to patient [3]. Therefore, it is important for periodontal treatment to target not only the disruption of the bacterial biofilm, but also to correct and improve the risk factors that have contributed to the disease’s onset and progress [4]. The significant influence of predisposing local and systemic factors for the pathogenesis of periodontal conditions is also highlighted in the 2018 classification [5]. From this new perspective, factors such as diabetes mellitus or smoking can severely aggravate the clinical manifestation and outcome in an affected patient. 

## 2. Materials and Methods

The search strategy for this narrative review consisted of four complementary directions for the writing of this paper: (i) basic principles of periodontal medicine; (ii) pathogenic interactions between periodontal diseases and diabetes mellitus; (iii) pathogenic interactions between periodontal and hepatic diseases (chronic hepatitis C, fatty liver disease) and (iv) pathogenic interactions between periodontal bacteria and gastrointestinal tract cancers. The search was performed within PubMed/Medline, Scopus, Google scholar and Science Direct databases. The MESH terms used during the search were “periodontal disease”, “periodontal medicine”, “periodontitis”, “diabetes mellitus”, “liver disease”, “chronic hepatitis C”, “steatosis”, “NAFLD”, “MAFLD”, “oral bacteria”, “oral cancer”, “periodontal pathogens”, “gastric cancer”, “colorectal cancer”, “pancreatic cancer”. Selected papers consisted of peer-reviewed and full-length English language articles (articles and reviews). Relevant studies were extracted and critically analyzed for inclusion in this review, resulting in information that was structured into sections. A section on the new classification of periodontal diseases was also included, given the high relevance and increased novelty of this topic. The information was organized in order to offer a comprehensive insight into the pathogenic connections of periodontitis and metabolic/digestive tract disorders, highlighting the impact that periodontal disease may have on the general homeostasis of the human body. 

## 3. The New Classification System of Periodontal Diseases

The 2017 World Workshop of the European Federation of Periodontology and of the American Academy of Periodontology laid the guidelines and framework for a new classification system of periodontal diseases, which was later published in 2018 [5]. The purpose of the workshop was to update and to adapt the previously existing classification [6] in light of the recent findings on periodontal disease pathogenic mechanisms and clinical features. Another key element was to include into the classification peri-implant inflammation (peri-implantitis), as implant dentistry has seen a major rise in popularity in the last decades. The joint efforts of European and American experts also targeted the difference among localized and generalized forms of gingival inflammation and the establishment of gingival bleeding on probing as a key clinical indicator of gingival disease [7]. Another innovative element of the new classification was to include the definition of periodontal health, both on an intact and on a reduced periodontium, with the latter referring to a patient who has undergone complete and successful periodontal therapy. Thus, it was generally accepted that a gingivitis patient may revert to an intact periodontal status, while a periodontitis patient suffers irreversible damage to their periodontal tissues, which will lead to a reduced periodontal status, even after being successfully treated. This implies that a patient with a reduced periodontium will require a constant and careful monitoring of their status [8].

During the twenty years that passed between the two latest classification systems (1999 and 2018) great scientific effort was put into fundamental and clinical research in periodontology [7]. This research has led to a better understanding of the pathogenic mechanisms and therapeutical protocols that are required by periodontology specialists. Using the concept of “evidence-based medicine”, the experts correlated the fundamental and clinical findings of the studies, concluding that periodontal diseases can be divided into three categories: (i) necrotizing periodontal disease, (ii) periodontitis as manifestation of systemic diseases and (iii) periodontitis. The third category (periodontitis) encompasses all previously known forms of “chronic” or “aggressive” periodontal disease. In order to improve the case definition and to provide an easily understandable and generally applicable diagnostic, a system of staging and grading was elaborated for periodontitis. The stages of periodontitis (from Stage I = Initial form, to Stage IV = Severe with extended teeth loss) refer to the severity of periodontitis, while the grading scale (Grade A = Slow, to Grade C = Rapid) refers to the rate of progression of the disease [5]. For example, the system implies that a patient with poorly controlled diabetes mellitus (glycated hemoglobin ≥ 7%) is given a Grade C periodontitis diagnosis, reflecting the rapid rate of progression of disease, regardless of other clinical periodontal parameters. This diagnosis algorithm is supported by the strong mutual influence exerted by diabetes and periodontitis. 

The staging of periodontitis offers practitioners valuable information on its severity but also on the complexity of the required therapeutical protocol, while the grading system allows the inclusion of periodontal biological characteristics into the diagnostic algorithm. Thus, the specialist can take into account the patient’s periodontal history when evaluating the risk of future disease progression, in order to assess whether the treatment may have an unsatisfactory result or whether the disease may have negative consequences on the systemic heath status. Therefore, the practitioner is allowed a more individualized and personalized approach to the treatment protocol, in order to increase its efficacy and the health-inducing effects on the patient’s general status [5,6,7,8]. Such treatment protocols could include novel approaches in the future, such as fibrin-neutrophil cell engagement disruptors [9]. 

## 4. The Concept of “Periodontal Medicine”

The periodontium and its structural components (gingiva, cementum, periodontal ligament and alveolar bone) are anatomical elements that possess a rich vascular and nervous network. This fact creates a strong bond between the periodontium and the rest of human body, via vascular, lymphatic and nervous means [10]. Therefore, any pathologic event that may influence the general homeostasis of the body and the health status of patients could be reflected at a periodontal level [10]. Conversely, periodontal disease and its treatment can modify the general health status of a patient and can influence the evolution of certain systemic diseases [11]. During the past decades, extended scientific research has led to the development of the concept of “periodontal medicine”, which comprises the bidirectional links that lay between periodontal and certain systemic conditions, including diabetes mellitus, cardiovascular diseases or rheumatoid arthritis [12,13].

The “periodontal medicine” concept was first introduced by Professor Steven Offenbacher, with his life-long research bringing an immensely valuable contribution to the holistic and comprehensive view of periodontal pathology and its systemic implications [13]. The concept of “periodontal medicine” describes an emerging field of periodontology, which focuses on the systemic implications of periodontal disease and whether periodontal therapy can also contribute to the compensation and improved control of specific conditions, mainly diabetes mellitus and cardiovascular diseases [13]. The concept also focuses on the impact of periodontal disease on pregnancy outcomes and whether a mother’s periodontal disease can influence premature birth or a low birth weight of the child [13].

The concept of “periodontal medicine” also has important public health implications. Periodontal disease can be easily diagnosed by performing a periodontal screening during regular visits to the dental office [14]. These regular dental visits can also be used for preventive measures, which ensure the disruption of the bacterial subgingival biofilm that causes periodontal disease [15]. Thus, through prevention or early diagnosis of periodontal disease, its significant future impact on systemic heath can also be decreased and controlled [16]. In addition, the dental practitioner can also take advantage of the patient’s visit to the dental office and ask them to undergo a series of standard blood tests, which are valuable for the assessment of their systemic heath, if the patient has not done this for a long period of time. Therefore, the regular visits of patients to the dental office can be used for preventive measures, both periodontal and systemic, which can help the early diagnosis and treatment of certain conditions, allowing favorable prognosis for their resolution [13]. 

With the official recognition of the “periodontal medicine” concept and the new 2018 classification of periodontal diseases, practitioners worldwide are able to better understand the systemic implications of periodontal conditions and the benefits that periodontal therapy has on the rebalancing of a disrupted homeostasis. Hence, periodontal evaluation and periodontal therapy should be viewed as an indispensable part not only of a good oral health, but of good health and well-being in general [17]. More recently, during the ongoing COVID-19 pandemic, periodontitis has been shown to significantly impact the outcome of SARS-CoV-2 infections in individuals with poor oral health [18]. 

## 5. Periodontitis and Diabetes Mellitus

Diabetes is one of the most common diseases and represents a complex and heterogeneous group of chronic metabolic diseases characterized by insufficient insulin secretion and/or an inefficiency of targeted tissues in terms of its metabolic action. Diabetes mellitus is a metabolic disease that can be broadly classified into type 1, type 2, and gestational diabetes. 

Type 1 diabetes occurs predominantly in people < 30 years old and is generally thought to be precipitated by an immune-associated destruction of insulin-producing beta cells in the pancreas, leading to insulin deficiency and requiring exogenous insulin supplementation. The development of beta cell autoantibodies is thought to be induced after a genetically susceptible individual is exposed to a presumed environmental factor that triggers a loss of immune regulation [19]. Destruction of beta cells leads to a decrease in insulin secretion, development of hyperglycemia, and ultimately clinical type 1 diabetes [20].

Type 2 diabetes comprises the vast majority of all diabetes cases in adults and is a progressive metabolic disease characterized by insulin resistance [21]. The pathogenesis of type 2 diabetes primarily initiates with the inadequacy of pancreatic islet beta cells to respond to chronic fuel surfeit, hence causing glycemic load, insulin resistance and obesity [22,23]. Pancreatic beta cell dysfunction is closely related with the initiation and progression of both type 1 and type 2 diabetes [23]. 

In pregnant women without any previous history of diabetes, the development of increased blood-sugar levels is responsible for gestational diabetes. Postpartum, most women revert to their prediabetic states, but are predisposed to the development of diabetes during the later stages of life. Women with gestational diabetes mellitus in whom glucose tolerance becomes normal postpartum remain insulin-resistant compared to women with no history of this type of diabetes [24]. The demographics of pregnant women have changed in recent decades, so that as the rate of women giving birth at an older age increase, so does the rate of obesity. This has led to an increase in the prevalence of gestational diabetes, establishing the disease as an imminent global concern [25].

### 5.1. Trends in Epidemiology of Diabetes 

The number of people with diabetes has increased from 108 million in 1980 to 450 million in 2021 and is considerably higher in middle- and lower-income countries [26]. Type 1 diabetes is one of the major autoimmune diseases developing in childhood, with the incidence rate ranging between 3 and 5% every year [27]. The incidence of type 1 diabetes increases with age, up to a peak around 10–14 years old, but the disease can occur at any age. According to some studies, the incidence tends to be higher in boys than in girls in high-incidence countries, with the opposite pattern seen in low-incidence countries [28]. Early indications of a steeper relative increase among young children are no longer seen. In the long term, most countries have shown non-linear changes, with periods of small or no increase, such as in Norway from 2004 to 2012 and in Finland from 2006 to 2011. A small increase in incidence was seen in the USA during 2002–2012 [29]. According to the reports by the International Diabetes Federation (IDF), 387 million people have diabetes, which is expected to rise to 592 million by 2035 [30].

The majority of diabetes is type 2, which generally follows a period of prediabetes, a condition where blood glucose levels are higher than normal, but not high enough for a type 2 diabetes diagnosis. Type 2 diabetes can be prevented or delayed through mitigation of modifiable risk factors, such as healthier eating, weight loss and increased physical activity [31].

According to The American Diabetes Association standard of diabetes care for patients and health care workers, diabetes care should include comprehensive medical assessment of comorbidities, lifestyle management, glycemic control, medication, obesity management, risk reduction and prevention of diabetes complications [32].

### 5.2. Bidirectional Relation between Diabetes and Periodontal Disease 

Systemic diseases, such as diabetes, can interfere with the periodontal condition, making the prognosis of associated diseases unfavorable [33]. The chronic evolution of inflammatory diseases is possible in complex conditions, because they often involve several causal components that play a simultaneous role and interact with each other, often in an unpredictable way. Thus, the disease results from complex interactions between genetic activity and the environment, such as bacterial communities (biofilms) and the host’s immune response [34].

Over the years, diabetes has been linked to nephropathy, retinopathy, neuropathy, cardiovascular disease and periodontitis. Periodontal disease, considered an inflammatory disease, has been shown to have a number of systemic implications, with several studies suggesting the existence of a two-way link between periodontal health and these various pathologies [33,34,35]. Over the years, essential evidence has shown that diabetes is a risk factor for impaired periodontal health. Studies that have focused on examining the relationship between diabetes and periodontal disease have shown an increase in incidence; this association that has increased scientific interest [36,37]. Patients with diabetes generally have a higher prevalence of periodontal disease than the healthy population, with a clear relationship between diabetes and different clinical conditions related to periodontal parameters. Tissue metabolic imbalances can reduce the resistance to infection of diabetic patients and thus can influence the initiation, development and progression of periodontal disease [38,39].

Certain cytokines and inflammatory mediators are involved in the association of the two diseases and appear to have a mutually harmful effect [40]. Given the important role of inflammatory processes in type-1 diabetes and knowing that periodontitis is associated with increased production of serum inflammatory markers, concentrated studies have been conducted to determine whether periodontal inflammation can influence metabolic control of type 1 diabetes [40]. Studies have found a strong correlation between periodontal disease and diabetes, as well as between periodontal disease and metabolic control of diabetes, in both type 1 and type 2 diabetes [41,42].

The inflammatory reaction is a basic feature of autoimmune disorders, but also of periodontal disease. Increased secretion of pro-inflammatory cytokines, including interleukin-1, interleukin-8, interleukin-6, tumor necrosis factor-alpha and C-reactive protein, during the development of periodontitis, may exert its effect by activating inflammatory pathways in patients with diabetes [43]. Increased expression of low-density lipoproteins (LDL), triglycerides and polyunsaturated fatty acids is present with decreased activity of enzymes such as 6-desaturase. This is caused by disruption of membrane proteins and the phospholipid layer of the cell membrane, leading to impaired cell function/homeostasis and healing. Hyperglycemia and hyperlipidemia lead to the formation of phenotypes of monocytes receptive to lipopolysaccharides [44], similar to that of *Porphyromonas gingivalis*.

Hyperlipidemia, which changes immune cell function, is associated with the up-regulation of pro-inflammatory cytokines in polymorphonuclear monocytes and leukocytes [45,46]. Interleukins such as interleukin-1beta cause an increase in prostaglandins and matrix metalloproteinase, with decreased collagen synthesis and up-regulation of T and B lymphocytes [47]. Tumor necrosis factor-alpha is associated with increased cellular apoptosis and bone resorption. It has also a downward regulation effect on macrophage growth factors [48].

Oxidative stress is a key factor that may explain the prevalence and severity of periodontitis in patients with diabetes. The activity of the prooxidative enzyme myeloperoxidase in the gingival crevicular fluid of periodontal patients with diabetes is low compared to that of patients without diabetes and patients with diabetes but without inflammatory damage of the periodontal tissue [49]. Patients diagnosed with a form of periodontal disease and diabetes showed an imbalance between pro-oxidants and antioxidants in the body, thus promoting cell damage and increasing the extent of the inflammatory response effects [40,50].

## 6. Periodontitis and Liver Diseases

Hepatic conditions, such as Chronic Hepatitis C (CHC) and Metabolic Associated Fatty Liver Disease (MAFLD), have an important inflammatory component and are driven by the up-regulated synthesis of certain pro-inflammation mediators. Though different by etiology (CHC–viral, MAFLD–metabolic), both conditions lead to the development of chronic liver inflammation. Fueled by the up-regulated cytokines, this hepatic inflammation replaces normal hepatic tissue with the fibrotic type (hence, the term liver fibrosis) [51]. Gradually, the liver fibrosis expands to such an extent that the liver can no longer perform its crucial systemic functions, thus leading to life-threatening complications such as liver cirrhosis, liver failure or liver carcinoma [52].

Though not life-threatening in itself, periodontitis can have significant connections with other major systemic diseases, as depicted by the “periodontal medicine” concept. The pathogenic mechanisms of periodontitis may be similar in some aspects to that of hepatic conditions, in that an exogenous factor (bacterial biofilm) triggers the onset of an inflammatory reaction, which consequently becomes chronic and causes the destruction of periodontal tissues [53]. This mechanism is also found in degenerative hepatic conditions (as CHC and MAFLD). The inflammatory reaction in both periodontitis and hepatic conditions is driven by the same pro-inflammatory mediators, thus generating the hypothesis that the two type of conditions may share a pathogenic connection [54,55].

The evaluation of the periodontal and dental status of periodontitis patients with CHC has shown that it was significantly more negatively modified than that of non-CHC periodontitis participants, in terms of tooth loss, number of periodontal pockets, periodontal pocket depth, clinical attachment loss and gingival index [55]. Furthermore, periodontitis + CHC patients exhibited significantly elevated gingival fluid levels of specifically-hepatic-synthetized enzymes, such as aspartate-aminotransferase and alanine-aminotransferase [56,57]. This shows that their modified hepatic activity can also be reflected by their periodontal status and gingival fluid composition. Regarding the pro-inflammatory mediator composition of the gingival fluid, it was shown that periodontitis + CHC patients had significantly up-regulated levels of interleukin-1alpha and -1beta compared to the non-CHC periodontitis group [56]. These two interleukins are key pro-inflammatory mediators, which trigger and fuel the inflammatory reaction in both periodontal and hepatic conditions. The gingival tissue samples of periodontitis + CHC patients also exhibited certain histological and imagistic structural changes that suggest the development of an exacerbated inflammatory gingival reaction [58,59]. These preliminary findings encourage the development of the study on a broader level, in order to reach more conclusive results. 

Concerning MALFD, though its etiology is different to that of periodontitis, the two conditions share similar risk factors, including obesity, type 2 diabetes mellitus and smoking [60]. Both diseases have also been linked to the pathologic process of insulin resistance, suggesting the possibility of a crossed interaction. The study of MAFLD + periodontitis patients has revealed that their periodontal status was significantly more negative in terms of periodontal clinical parameters than that of non-MAFLD patients with periodontitis, suggesting a more severe periodontal breakdown in these patients [61]. Moreover, their metabolic parameters, such as glycemia, cholesterol levels and body-mass index, were significantly increased compared to those of MAFLD patients with no periodontitis, suggesting that periodontal disease may have an additional negative effect on the general homeostasis of MAFLD patients. Together with these initial results, further studies on specific pro-inflammatory mediators and insulin-resistance mechanisms may improve the understanding of possible connections between periodontitis and MAFLD [60].

## 7. Periodontal Pathogens and Digestive Tract Cancers

It is known that the oral health condition and especially the periodontal status of the patient is in direct relationship with the composition of the oral microbiome [62]. Bacteria from the saliva are constantly swallowed, and they are in permanent contact with the mucosa of the digestive tract, even though the distances that the bacteria have to travel to each organ are different. Multiple studies have associated the oral microbiota, and especially the periodontal pathogens like *Porphyromonas gingivalis*, with various gastrointestinal diseases, showing a higher bacterial diversity in patients with gastro-intestinal cancers [63,64]. *Tannerella forsythia* is another bacterium involved in oral diseases, which has been found in gastrointestinal cancers [65,66]. Moreover, important differences between the oral microbiome of patients with colorectal cancer and the ones with upper digestive tract cancer were highlighted [65]. It is estimated that in 2018 alone there were more than 4 million newly diagnosed cases of digestive tract cancers worldwide, including colorectal, gastric, esophageal, pancreatic and oral cancer, and that more than 2 million patients died as a result [67]. Although research in this field is just at the beginning, the oral cavity and saliva could be used for more detailed investigations and for detecting digestive tract cancers without using other invasive methods (see Table 1).

Regarding oral inflammation, when measuring the bleeding on probing, patients with colorectal, esophageal, tongue and pharyngeal cancer had higher values. The patients with tongue cancer had more decayed teeth than the control group, while the gastric cancer patients had fewer than the control group. In addition, the overall oral hygiene status was better in gastric and colorectal patients [63].

It has been postulated that the real reason for oncogenesis in the digestive tract is the biofilm and not the bacteria themselves, even though several bacteria like *Fusobacterium nucleatum*, *Bacteroides fragilis* and *Streptococcus gallolyticus* have been incriminated [68]. Armstrong et al. showed that chronic bacterial infection leads to chronic inflammation and eventually to the development or progression of cancer [69]. Moreover, another study shows that about 15% of cancers from all over the world can be linked with chronic infections [70]. Oral *Klebsiella* colonizes the intestinal mucosa, causing chronic inflammation [71]. Pathogens like *Fusobacterium nucleatum* and *Streptococcus gallolyticus* often cause infections associated with biofilms, increasing virulence and causing major changes in the host immunity, such that they are implicated as possible etiological factors for the development of colorectal cancer [63,68,72]. The link between the bacterial microorganisms and oncogenesis is different for each organ of the digestive tract, as each organ has its own distinct microbiome [73].

*Porphyromonas gingivalis* was found in higher levels in the saliva of patients who had digestive tract cancers, compared with the control group. In tongue, pharyngeal and esophageal cancer, the salivary microbiome is more complex, consisting mostly of *Fusobacterium nucleatum, Streptococcus parasanguinis II* and *Neisseria*. In gastric cancer, low levels of *Corynebacterium* and high levels of *Neisseria* were identified, whereas the salivary microbiome of patients with colorectal cancer was more abundant in *Actinomyces odontolyticus. Prevotella melaninogenica, Porphyromonas pasteri* and *Streptococcus* species were found in higher levels in cancer-free patients [63]. 

The immunity of the host can be impaired by periodontitis, which also leads to the alteration of the complexes of bacteria in the oral environment as well as the complexes from distal sites, which can lead to an abnormal host response [72]. Considering this point, the localized inflammation of the periodontal structures could also be linked to the inflammation of distal sites, such as in inflammatory bowel disease, as highlighted by a recent review on the topic [65]. 

### 7.1. Oral Cancer

Periodontal pathogens like *Porphyromonas gingivalis, Treponema denticola* and *Fusobacterium nucleatum* have been incriminated in the genesis and evolvement of oral cancer, while oral *Streptococci* decrease T-cell responses and provide anti-tumor immunity [73,74,75]. In one study, Chang et al. found higher levels of *Porphyromonas gingivalis* in samples of oral squamous cell carcinoma and also stated that this can be linked with lymph node metastasis, poor differentiation and late cancer staging [76]. It is thought that it promotes oncogenesis by suppression of the immune function, triggering chronic inflammation, increasing cell invasion and proliferation and decreasing cell apoptosis [75]. *Fusobacterium nucleatum* has been found in high levels in tissues and saliva of patients with oral squamous cell carcinoma, compared to the control cases [77]. Impairment of the immune function and maintaining of chronic inflammation have been incriminated as possible mechanisms for the development of oral cancer [75]. Another periodontal pathogen that has been linked with oral squamous cell carcinoma and oropharyngeal squamous cell carcinoma is *Treponema denticola* [74,78].

### 7.2. Colorectal Cancer

Specific biofilms were found on colorectal tumors within normal surgical margins and in genetically predisposed individuals with familial adenomatous polyposis, compared to healthy individuals, in whom these bacteria could not be found. The biofilm abounded in *Bacteroides fragilis* and periodontal pathogens like *Fusobacterium nucleatum* and *Peptostreptococcus stomatitis* [79,80]. Moreover, a recent study links fusobacteria with pancreatic cancer [81].

There are numerous studies that link *Fusobacterium nucleatum* with colorectal cancer, predominantly proximal colon tumors [82], including Komyia et al., who found the oral strain of fusobacteria grafted on the colorectal carcinoma [83]. *Fusobacterium nucleatum* has been shown to bind to oncogenic cells through its protein, Fap2, and to favor the development of colorectal cancer [84]. *Fusobacterium nucleatum* binds to numerous bacteria, with almost all species involved in oral plaque formation [85]; the real reason behind oncogenesis may be the biofilm that *Fusobacteria* gather, by co-aggregating with the other species, and not the bacterium itself [86]. *Fusobacterium nucleatum* and *Porphyromonas gingivalis* help each other survive in the oral environment, and it is suggested that they can do the same within the intestinal microbiome, triggering tumorigenesis [87]. *Streptococcus gallolyticus*, formerly known as *S. bovis*, was also incriminated in colorectal oncogenesis [88].

### 7.3. Gastric Cancer

One of the most hostile environments of the human body is the gastric one, with its low pH that kills most bacteria. *Helicobacter pylori* is one of the few bacteria that can survive in these conditions, probably because of its urease activity, which overcomes gastric acid [89]. It localizes deep in the glands, in direct contact with the stem cells, at the bottom of the glands, or in the region of the neck, where there is a continuous proliferation of the cells [90]. A study showed that in mouse models infected with *Helicobacter pylori*, the intraepithelial neoplastic tissue appeared much earlier in mice with a more complex gastric microbiota than ones without the commensal bacteria [91]. 

Other bacteria that have been found grafted on gastric tissue are *Streptococcus*, *Lactobacillus*, *Rothia*, *Prevotella*, *Veilonella*, *Neisseria* and *Haemophilus* [92]. Some authors have suggested that the interactions between *Helicobacter pylori* and the other bacteria forming the gastric microbiota may be a triggering factor for oncogenesis [68]. There are studies showing that mice, five months after being infected with both *Helicobacter pylori* and *Streptococcus salivarius*, developed severe inflammation, dysplasia and hyperplasia in the gastric tissue [93]. Other periodontal pathogens, like *Treponema denticola* and *Aggregatibacter actinomycetemcomitans*, have been incriminated in the development of pre-cancerous lesions [94].

### 7.4. Pancreatic Cancer

High levels of *Neisseria elongata* and *Streptococcus mitis* were found in the microbiota of pancreatic cancer patients, thus allowing detection of pancreatic cancer with a 96.4% sensitivity and 82% specificity. Moreover, after analyzing a model with the combination of *Porphyromonas*, *Leptotrichia*, *Haemophilus* and *Fusobacterium*, the researchers were able to distinguish between pancreatic cancer patients and the control group [95].

## 8. Conclusions

The assessed papers are rich in content connecting periodontitis to the targeted systemic diseases. While for some pathologies (such as diabetes mellitus), the shared pathogenic mechanisms are strong and offer the perspective of mutual bi-directional influence, for other metabolic/digestive disorders, future research is required in order to reach similar conclusions. For the moment, the connections between periodontal and hepatic conditions seem to be driven by the shared pro-inflammatory mediators, while the link between periodontitis and gastric-tract cancer could have a bacterial justification. This motivates future research on these topics, which would enable a better understanding of their probably mutually shared pathogenic mechanisms. 

## Figures and Tables

**Table 1:** Possible involvement of periodontal pathogens in the onset of gastrointestinal cancers.

Periodontal Pathogen	Type of Gastrointestinal Cancer			
	Oral Cancer	Colorectal Cancer	Gastric Cancer	Pancreatic Cancer
*Porphyromonas gingivalis*	*	*		*
*Treponema denticola*	*		*	
*Fusobacterium nucleatum*	*	*		*
*A. actinomycetemcomitans*			*	

* relevant existing scientific data.

## Data Availability

Not applicable.
